# Supplementary material for: Rapid and definitive treatment of phenylketonuria in variant-humanized mice with corrective editing
Source: Nat Commun. 2023 Jun 10;14:3451. doi: 10.1038/s41467-023-39246-2 (PMC10257655; doi:10.1038/s41467-023-39246-2)
Supplement: Supplementary file 1 — Supplementary Information [file 41467_2023_39246_MOESM1_ESM.pdf]

Supplementary Table 1. Assessment of off-target editing with ABE8.8/PAH2 with plasmid delivery in HuH-7 cells.

Genomic site	Protospacer/PAM sequence*	Amplicon (chromosome: position range)†	ONE-seq scores‡	Treated sample 1§	Treated sample 2	Control sample 1	Control sample 2	Mean net A→G editing %
PAH	CACAGTTCGGGGGTATACATGGG	chr12:102852743+102852906	1.00 / 1.00	54.44	55.28			
OT1	CACAGTaCGGtGGTATACATCTG	chr2:26058450-26058634	2.18 / 2.01	0.20	0.30	0.03	0.02	**+0.23**
OT2	tACAGTTCaGGaGTATACATCTG	chrX:25828488+25828614	1.27 / 0.99	0.04	0.03	0.03	0.04	+0.00
OT3	CACAGcTCaGaGGTATcCATAGA	chr19:4229767+4229891	1.04 / 0.89	0.02	0.03	0.05	0.05	–0.02
OT4	CACAG-TgGGGGtTATACAgTGG	chr6:124527035-124527205	0.93 / 0.63	0.03	0.03	0.02	0.03	+0.00
OT5	CACAG-TaGGaaGaATACATGGG	chr9:22691618-22691742	0.82 / 0.68	0.05	0.07	0.08	0.06	–0.01
OT6	tACAG-TCaGGGGTAgACATAGA	chr10:32696053+32696242	0.76 / 0.41	0.04	0.03	0.03	0.04	+0.00
OT7	CACAGTTa-GGGGTATACcTGTG	chr2:26246797-26246966	0.71 / 0.59	0.03	0.10	0.05	0.05	+0.02
OT8	tACAGTTCataGGTATACATATA	chr15:56186524-56186749	0.57 / 0.51	0.04	0.04	0.03	0.03	+0.01
OT9	CACAGTTC-ctGGTATACAgTAG	chr1:45040620-45040761	0.52 / 0.32	0.02	0.03	0.02	0.02	+0.00
OT10	CACAGcTC-GaGcTATACATTGG	chr5:125457800-125457997	0.41 / 0.26	n.d.	n.d.	n.d.	n.d.	n.d.
OT11	CACAG-TaGGaGGTgaACATGGG	chr6:150192467-150192579	0.40 / 0.34	0.04	0.04	0.04	0.04	+0.00
OT12	CACAG-TaGGtGGTATAtATTGG	chr4:149160588+149160738	0.40 / 0.26	0.05	0.08	0.06	0.03	+0.02
OT13	CACAGTataGaGGaATACATGTG	chr3:45828272-45828471	0.40 / 0.36	0.05	0.03	0.04	0.03	+0.00
OT14	aACAGTTCaGaGGTAgACATGGG	chr8:97435202+97435304	0.39 / 0.32	0.11	0.07	0.04	0.04	+0.05
OT15	CACAG-TaGGaGGTAaACAgTGG	chr6:111583293+111583460	0.37 / 0.32	0.11	0.04	0.05	0.07	+0.02
OT16	CACAGTTa-GGaGTATAtATATG	chr14:28196330-28196500	0.35 / 0.28	0.01	0.01	0.04	0.03	–0.03
OT17	CACAagTCGGaGtTATACATTGA	chr5:29693247-29693418	0.33 / 0.31	0.02	0.03	0.05	0.03	–0.01
OT18	CACAGTTCaGaGGTATACtcTGA	chr1:169997464+169997610	0.26 / 0.26	0.07	0.06	0.02	0.04	+0.04
OT19	CACAaTTa-GaGGTATACATTTG	chr3:85844516-85844764	0.26 / 0.18	n.d.	n.d.	n.d.	n.d.	n.d.
OT20	CACAGcT-GGGGGTATgCATGGG	chr10:87939169-87939267	0.25 / 0.24	8.17	8.76	0.01	0.02	**+8.45**
OT21	CACAGTTCaGaGtATATACAgAGG	chr9:24491371-24491521	0.24 / 0.18	0.03	0.04	0.03	0.04	+0.00
OT22	CACAG-TataGGGTATAtATGGG	chr11:10220712-10220849	0.21 / 0.13	0.07	0.11	0.04	0.05	+0.04
OT23	CACAGaTa-GGaGTATACATTGA	chr11:122993134-122993239	0.18 / 0.12	0.11	0.10	0.04	0.03	+0.07
OT24	CACAGTTC-aGGGTAgACATGGA	chr3:139094035+139094192	0.18 / 0.16	0.05	0.06	0.05	0.05	+0.01
OT25	CACAG-TaGGaGGTATACAaATG	chr6:44087717-44087854	0.18 / 0.18	0.04	0.08	0.04	0.07	+0.00
OT26	aACAGaTCaGtGGTATACATGGA	chr10:116338730+116338928	0.18 / 0.11	0.08	0.13	0.17	0.11	–0.04
OT27	CACtaTT-GGGaGTATACATTAG	chrX:54847574+54847732	0.17 / 0.13	n.d.	n.d.	n.d.	n.d.	n.d.
OT28	CACAGTcaaaaGGTATACATAGC	chr13:90190890-90191003	0.17 / 0.12	0.07	0.05	0.05	0.05	+0.01
OT29	CACAGTTgGGGaG-ATACAgTGG	chr2:29883422-29883580	0.14 / 0.12	0.01	0.03	0.02	0.02	+0.00
OT30	aACAGTaCaGaGGTATACAgGGT	chr14:23897506-23897740	0.13 / 0.10	0.08	0.11	0.07	0.08	+0.02
OT31	CACAG-TCaGaGGTATACAcTGC	chr20:45189314-45189457	0.13 / 0.12	0.05	0.06	0.07	0.07	–0.01
OT32	tACAGTcCaaGGaTATACAgTGG	chr12:75897600+75897756	0.13 / 0.09	0.04	0.05	0.05	0.04	+0.00
OT33	CACAGTagGtaGGaATACATGGG	chr22:41123474+41123609	0.13 / 0.10	0.03	0.02	0.04	0.03	–0.01
OT34	CACAGTTCtGGaGTAaACATCAG	chr14:72798857+72798995	0.12 / 0.11	0.02	0.03	0.03	0.03	+0.00
OT35	CACAG-cCtGaGGTgTACATTGG	chr4:82317915-82318094	0.12 / 0.11	n.d.	n.d.	n.d.	n.d.	n.d.
OT36	CACAtaTCaGGtGTATACATACG	chr3:175369505-175369666	0.11 / 0.09	n.d.	n.d.	n.d.	n.d.	n.d.
OT37	CACAG--aGGGGaTAcACATGTG	chr8:138860206-138860394	0.10 / 0.08	0.04	0.04	0.01	0.05	+0.01
OT38	CACAGgT-GGGGcTAaACATTGG	chrX:89246615-89246810	0.10 / 0.06	n.d.	n.d.	n.d.	n.d.	n.d.
OT39	aACAaTTCaGaGGTATACATAGA	chr1:167995651+167995832	0.09 / 0.06	n.d.	n.d.	n.d.	n.d.	n.d.
OT40	CACAGTaC-aGGGTAgACtTGGG	chr7:24877740+24877893	0.09 / 0.11	0.07	0.04	0.05	0.06	+0.00
OT41	CACAGTTtGGaaGTATACATCAA	chr4:170964177+170964341	0.09 / 0.08	0.02	0.01	0.01	0.03	+0.00
OT42	CACAGTTCaGGGGTTgTACATTAG	chr17:28871219+28871417	0.09 / 0.08	0.03	0.06	0.05	0.04	+0.00
OT43	CACAGTgaGGGGtAgATACATGGG	chr15:87616852+87617003	0.08 / 0.07	0.06	0.06	0.03	0.07	+0.01
OT44	CACAGTTCaGGaGTcTACtaAGG	chr13:73318393-73318587	0.08 / 0.08	n.d.	0.03	0.05	0.05	–0.02
OT45	CACAGTTCGaaGGTATgCtcAGG	chr9:90943419-90943575	0.07 / 0.07	0.02	0.02	0.04	0.04	–0.02
OT46	CACAGTT-GaGGGTgTgCATTGA	chr2:14561507+14561662	0.07 / 0.09	0.04	0.03	0.05	0.04	–0.01
OT47	CACAG-TCGGaGGTAgACAgGGC	chr18:52175622+52175732	0.07 / 0.08	0.04	0.03	0.04	0.04	–0.01

*The *PAH* on-target sequence and top ONE-seq-nominated candidate off-target sequences with mismatched positions and bulges indicated by lower case letters and dashes.

†Coordinates in the GRCh38/hg38 assembly of the human genome; "+" and "-" indicate forward or reverse orientation of protospacer/PAM sequence.

‡ONE-seq scores for duplicate experiments shown, with the table rank-ordered by the scores for one of the experiments.

§n.d. = PCR was unsuccessful for the genomic site.

Supplementary Table 2. Assessment of off-target editing with ABE8.8/PAH1 with plasmid delivery in HuH-7 cells.

Genomic site	Protospacer/PAM sequence*	Amplicon (chromosome: position range)†	ONE-seq scores‡	Treated sample 1§	Treated sample 2	Control sample 1	Control sample 2	Mean net A→G editing %
PAH	TCACAGTTCGGGGGTATACATGG	chr12:102852743+102852906	1.00 / 1.00	62.67	61.81			
OT1	TCACAGTTCGaaGGTAacaAGGC (repeat sequence in *LPA* locus)	chr6:160642695-160642814<br>chr6:160637152-160637271<br>chr6:160631603-160631722<br>chr6:160626059-160626178<br>chr6:160620513-160620632<br>chr6:160614961-160615080	1.77 / 1.20<br>1.76 / 1.22<br>1.41 / 1.02<br>1.39 / 0.96<br>0.82 / 0.65<br>0.63 / 0.54	0.02	0.03	0.03	0.03	–0.01
OT2	TCACAcTTCatGGGaATACAAGG	chr18:48720837+48721036	0.83 / 0.63	0.03	0.05	0.05	0.04	+0.00
OT3	aCACAGTcaGcGGGTATAaAAGG	chr2:10084715+10084871	0.81 / 0.91	0.06	0.05	0.07	0.05	+0.00
OT4	TCACAGTgaGGGGGTATgCAGGA	chr7:23126609-23126741	0.72 / 1.55	0.06	0.04	0.07	0.05	–0.01
OT5	TCACAG-TCGGaGGTAgACAGGG	chr18:52175632+52175760	0.69 / 1.19	n.d.	n.d.	n.d.	n.d.	n.d.
OT6	aCACAGTcCtGtGGTATACtTGG	chr7:131989636+131989782	0.68 / 0.92	0.08	0.04	0.04	0.05	+0.02
OT7	TCACAGcTCGGtGGaATACAGGG	chr1:118122495-118122686	0.66 / 0.83	0.15	0.15	0.04	0.04	**+0.11**
OT8	TCACAGTTaGtGGGaATACATGA	chr13:55307828+55307986	0.64 / 1.16	0.04	0.05	0.06	0.03	+0.00
OT9	TCACAGTTgaGGGG-ATACtGGG	chr8:111114094-111114285	0.60 / 1.96	0.06	0.04	0.07	0.04	+0.00
OT10	aCACAGTTCGaaGGTATgCtCAG	chr9:90943444-90943575	0.58 / 0.93	0.03	0.04	0.02	0.01	+0.01
OT11	cCACAGTTC-aGGGTAgACATGG	chr3:139094029+139094208	0.51 / 0.68	0.07	0.04	0.05	0.03	+0.02
OT12	aCACAGTcCtGtGGTATACAGAG	chr11:88649843+88650034	0.51 / 0.88	0.03	0.02	0.03	0.03	+0.00
OT13	TCAtAGTcCaGGGGTAcAtATGG	chr1:147746621-147746780	0.46 / 0.68	0.03	0.03	0.03	0.04	+0.00
OT14	TCACAGTTCaaaGGTA-ACAAGG	chr6:160609412-160609554	0.41 / 0.18	0.16	0.11	0.15	0.14	–0.01
OT15	TCACAGTTCaGGaGgATgCAAGG	chr8:29581469+29581662	0.40 / 0.59	0.06	0.05	0.02	0.03	+0.02
OT16	TCACAGTTgaGaGGTATACtGGC	chr4:68627582-68627777	0.37 / 0.72	n.d.	n.d.	n.d.	n.d.	n.d.
OT17	cCACAGTT-GGGaaTATACtGGG	chr4:16086757-16086918	0.34 / 0.74	0.04	0.02	0.08	0.00	–0.01
OT18	TCACAGTTgaGaGGTATACtGGC	chr4:68516236-68516505	0.33 / 0.69	0.07	0.06	0.03	0.03	+0.03
OT19	TCAtAGTcCtGGGGTATtCATAG	chr2:201571959-201572222	0.28 / 0.36	0.00	0.00	0.02	0.03	–0.03
OT20	TCACAG--CtGGaGgtTACAGGG	chr1:14673072+14673218	0.27 / 0.22	0.02	0.04	0.01	0.02	+0.01
OT21	gCAtAGTcCaGGaGTATAtATGG	chr4:189132563+189132698	0.26 / 0.34	0.03	0.03	0.03	0.04	–0.01
OT22	TCACAGTagGaGGGTATAaAAGG	chr14:106525120+106525331	0.25 / 0.87	0.05	0.02	0.05	0.05	–0.01
OT23	cCACAGTTCGGGGacATgCAGGG	chr7:158769358-158769517	0.24 / 0.45	0.01	0.01	0.03	0.03	–0.02
OT24	aCACAG-TCaGaGGTATACACTG	chr20:45189317-45189474	0.24 / 0.59	0.09	0.02	0.07	0.04	+0.00
OT25	aCACAGTaaGGtGGTATACtGGG	chr8:91553304+91553489	0.24 / 0.63	0.04	0.03	0.07	0.06	–0.03
OT26	TCACAGTTCtGGaGgATAtAGGG	chr7:45929162-45929294	0.24 / 0.44	n.d.	n.d.	n.d.	n.d.	n.d.
OT27	TCACAGTTCaGaGGgATcCAGGG	chr21:32427999-32428193	0.21 / 0.38	0.06	0.04	0.05	0.04	+0.00
OT28	TCAtAGTcCaaaaGTATACAGGG	chr5:22532873+22533087	0.20 / 0.28	0.01	0.00	0.02	0.01	–0.01
OT29	aCACAGTTCaGaGGTATACtCTG	chr1:169997458+169997557	0.19 / 0.64	0.05	0.04	0.04	0.06	+0.00
OT30	aCACAGTcCaGGGGaATAaAAGG	chr4:140189816+140189966	0.18 / 0.40	0.06	0.05	0.06	0.04	+0.00
OT31	cCACAGTTCaGGGGTAatCAAGG	chr17:48796646-48796843	0.18 / 0.48	0.05	0.04	0.03	0.06	+0.00
OT32	aCACAGTaCaGGGGaATACAGGC	chr11:15627919-15628082	0.17 / 0.43	0.07	0.05	0.03	0.03	+0.03
OT33	TCACAG-TgGGGGtTATACAGTG	chr6:124527035-124527205	0.16 / 0.47	0.03	0.04	0.01	0.02	+0.01
OT34	TCAtAGTTCGGGGaAaATACAAAG	chr3:188530545-188530676	0.14 / 0.69	0.02	0.03	0.02	0.01	+0.01
OT35	aCACAGTTCaaaGaTATcCAGGG	chr9:110553565+110553692	0.14 / 0.24	0.05	0.05	0.04	0.06	+0.00
OT36	TCACAGTTCGGtGGTATtgcAAG	chr9:21334697-21334807	0.14 / 0.15	0.03	0.04	0.02	0.03	+0.01
OT37	aCACAGTTCtGGGGTATAtACTG	chr11:126344469+126344632	0.14 / 0.21	0.04	0.07	0.06	0.05	+0.00
OT38	TCACAGTcCaGAGGGTATtaAGGG	chr5:20102259-20102434	0.14 / 0.22	0.04	0.05	0.03	0.05	+0.00
OT39	TCACAGTagGtaGGaATACATGG	chr22:41123444+41123591	0.13 / 0.24	0.02	0.05	0.05	0.02	+0.00
OT40	TCACAGTataaGGGaATACACAG	chr7:18116290-18116563	0.13 / 0.38	0.04	0.05	0.04	0.06	+0.00
OT41	TCACAG-TaGGaaGaATACATGG	chr9:22691618-22691742	0.12 / 0.20	n.d.	n.d.	n.d.	n.d.	n.d.
OT42	TCACAG--aaGGGGTATACAATG	chr6:61036724+61036870	0.12 / 0.56	0.04	0.04	0.05	0.03	+0.00
OT43	cCACAGTTaGGGGa-ATACtGGG	chr16:19630130+19630295	0.11 / 0.31	0.05	0.03	0.03	0.03	+0.01
OT44	TCACAGT--GGGtGTATACAGTG	chr8:93663922+93664104	0.11 / 0.33	0.03	0.02	0.04	0.02	–0.01
OT45	TCACAGT--aGGGcTATACACAG	chr11:88892985+88893143	0.10 / 0.29	0.05	0.06	0.07	0.07	–0.02
OT46	TCACAGT--GGaGGaATAtATGG	chr4:138339528-138339643	0.07 / 0.31	0.03	0.03	0.06	0.05	–0.02
OT47	TCACAGTcCGGaGGaATACtGGT	chr4:66261750+66261853	0.06 / 0.23	0.02	0.02	0.02	0.02	+0.00

*The *PAH* on-target sequence and top ONE-seq-nominated candidate off-target sequences with mismatched positions and bulges indicated by lower case letters and dashes.

†Coordinates in the GRCh38/hg38 assembly of the human genome; "+" and "-" indicate forward or reverse orientation of protospacer/PAM sequence.

‡ONE-seq scores for duplicate experiments shown, with the table rank-ordered by the scores for one of the experiments.

§n.d. = PCR was unsuccessful for the genomic site.

Supplementary Table 3. Assessment of off-target editing with ABE8.8/PAH1 with LNP delivery in HuH-7 cells.

Genomic site	Protospacer/PAM sequence*	Treated sample 1†	Treated sample 2	Treated sample 3	Control sample 1	Control sample 2	Control sample 3	Mean net A→G editing %
PAH	TCACAGTTCGGGGGTATACATGG	99.91	99.82	98.36				
OT1	TCACAGTTCGaaGGTAacaAGGC	0.03	0.03	0.02	0.03	0.03	0.03	+0.00
OT2	TCACAcTTCatGGGaATACAAGG	0.04	0.07	0.04	0.04	0.07	0.05	+0.00
OT3	aCACAGTcaGcGGGTATAaAAGG	0.08	0.06	0.09	0.10	0.08	0.10	–0.02
OT4	TCACAGTgaGGGGGTATgCAGGA	0.08	0.05	0.07	0.06	0.04	0.06	+0.01
OT5	TCACAG-TCGGaGGTAgACAGGG	n.d.	n.d.	n.d.	n.d.	n.d.	n.d.	n.d.
OT6	aCACAGTcCtGtGGTATACtTGG	0.05	0.07	0.05	0.04	0.02	0.03	+0.03
OT7	TCACAGcTCGGtGGaATACAGGG	0.13	0.23	0.15	0.03	0.04	0.04	**+0.13**
OT8	TCACAGTTaGtGGGaATACATGA	0.04	0.06	0.07	0.06	0.05	0.03	+0.01
OT9	TCACAGTTgaGGGG-ATACtGGG	0.06	0.03	0.05	0.10	0.04	0.00	+0.00
OT10	aCACAGTTCGaaGGTATgCtCAG	0.04	0.03	0.04	0.05	0.03	0.04	+0.00
OT11	cCACAGTTC-aGGGTAgACATGG	0.05	0.04	0.08	0.05	0.07	0.08	–0.01
OT12	aCACAGTcCtGtGGTATACAGAG	0.03	0.04	n.d.	0.03	0.01	0.06	+0.00
OT13	TCAtAGTcCaGGGGTAcAtATGG	0.06	0.04	0.03	0.04	0.03	0.05	+0.00
OT14	TCACAGTTCaaaGGTA-ACAAGG	0.27	0.34	0.19	0.40	0.26	0.45	–0.10
OT15	TCACAGTTCaGGaGgATgCAAGG	0.04	0.04	0.05	0.06	0.06	0.05	–0.02
OT16	TCACAGTTgaGaGGTATACtGGC	n.d.	n.d.	n.d.	n.d.	n.d.	n.d.	n.d.
OT17	cCACAGTT-GGGaaTATACtGGG	0.11	0.13	0.09	0.10	0.07	0.08	+0.03
OT18	TCACAGTTgaGaGGTATACtGGC	0.04	0.01	0.03	0.03	0.05	0.04	–0.01
OT19	TCAtAGTcCtGGGGTATtCATAG	0.02	0.07	0.03	0.00	0.00	0.05	+0.02
OT20	TCACAG--CtGGaGgtTACAGGG	0.02	0.02	0.02	0.02	0.03	0.04	–0.01
OT21	gCAtAGTcCaGGaGTATAtATGG	0.03	0.03	0.03	0.04	0.04	0.05	–0.01
OT22	TCACAGTagGaGGGTATAaAAGG	0.07	0.10	0.08	0.08	0.08	0.05	+0.01
OT23	cCACAGTTCGGGGacATgCAGGG	0.02	0.03	0.02	0.03	0.02	0.05	–0.01
OT24	aCACAG-TCaGaGGTATACACTG	0.06	0.07	0.05	0.00	0.11	0.10	–0.01
OT25	aCACAGTaaGGtGGTATACtGGG	0.04	0.05	0.05	0.06	0.07	0.04	–0.01
OT26	TCACAGTTCtGGaGgATAtAGGG	n.d.	n.d.	n.d.	n.d.	n.d.	n.d.	n.d.
OT27	TCACAGTTCaGaGGgATcCAGGG	0.05	0.09	0.05	0.06	0.05	0.07	+0.00
OT28	TCAtAGTcCaaaaGTATACAGGG	0.04	0.06	0.05	0.06	0.04	0.05	+0.00
OT29	aCACAGTTCaGaGGTATACtCTG	0.08	0.06	0.06	0.06	0.07	0.05	+0.01
OT30	aCACAGTcCaGGGGaATAaAAGG	0.07	0.06	0.08	0.07	0.06	0.07	+0.00
OT31	cCACAGTTCaGGGGTAatCAAGG	0.04	0.04	0.09	0.05	0.08	0.03	+0.00
OT32	aCACAGTaCaGGGGaATACAGGC	0.05	0.10	0.12	0.03	0.11	0.07	+0.02
OT33	TCACAG-TgGGGGtTATACAGTG	0.02	0.04	0.03	0.04	0.02	0.03	+0.00
OT34	TCAtAGTTCGGGGaAaATACAAAG	0.03	0.03	0.04	0.02	0.04	0.02	+0.00
OT35	aCACAGTTCaaaGaTATcCAGGG	0.05	0.06	0.06	0.05	0.06	0.07	+0.00
OT36	TCACAGTTCGGtGGTATtgcAAG	0.02	0.01	0.04	0.03	0.05	0.04	–0.01
OT37	aCACAGTTCtGGGGTATAtACTG	0.05	0.08	0.04	0.03	0.06	0.03	+0.01
OT38	TCACAGTcCaGAGGGTATtaAGGG	0.05	0.05	0.05	0.04	0.05	0.06	+0.00
OT39	TCACAGTagGtaGGaATACATGG	0.05	0.02	0.03	0.06	0.04	0.04	–0.01
OT40	TCACAGTataaGGGaATACACAG	0.06	0.07	0.07	0.06	0.05	0.06	+0.01
OT41	TCACAG-TaGGaaGaATACATGG	n.d.	n.d.	n.d.	n.d.	n.d.	n.d.	n.d.
OT42	TCACAG--aaGGGGTATACAATG	0.03	0.06	0.02	0.06	0.06	0.05	–0.02
OT43	cCACAGTTaGGGGa-ATACtGGG	0.06	0.03	0.09	0.10	0.04	0.07	–0.01
OT44	TCACAGT--GGGtGTATACAGTG	0.04	0.04	0.02	0.03	0.03	0.02	+0.00
OT45	TCACAGT--aGGGcTATACACAG	0.10	0.14	0.09	0.12	0.09	0.22	–0.03
OT46	TCACAGT--GGaGGaATAtATGG	0.07	0.02	0.06	0.04	0.03	0.02	+0.02
OT47	TCACAGTcCGGaGGaATACtGGT	0.02	0.03	0.03	0.02	0.03	0.05	–0.01

*The *PAH* on-target sequence and top ONE-seq-nominated candidate off-target sequences with mismatched positions and bulges indicated by lower case letters and dashes.

†n.d. = PCR was unsuccessful for the genomic site.

Supplementary Table 4. PCR primers for next-generation sequencing.

Site*	Forward primer	Reverse primer
PAH (human)	TCGTCGGCAGCGTCAGATGTGTATAAGAGACAG ACCTCATTCTTGCAGCAGGA	GTCTCGTGGGCTCGGAGATGTGTATAAGAGACAG TCTCGGGATTTCTTGGGTGG
PAH (mouse)	TCGTCGGCAGCGTCAGATGTGTATAAGAGACAG TGCTGGCTTACTGTCGTCTC	GTCTCGTGGGCTCGGAGATGTGTATAAGAGACAG GAGCATCCATTGTGGTTGGC
PAH1 OT1	TCGTCGGCAGCGTCAGATGTGTATAAGAGACAG GGGATCATATAGGGAATTTCGGA	GTCTCGTGGGCTCGGAGATGTGTATAAGAGACAG TCCAGTTTCTCGTGACAGGT
PAH1 OT2	TCGTCGGCAGCGTCAGATGTGTATAAGAGACAG AGAGAGTGGCCCTGTTGAG	GTCTCGTGGGCTCGGAGATGTGTATAAGAGACAG TCTGCCAAAATGAGCTGTAGG
PAH1 OT3	TCGTCGGCAGCGTCAGATGTGTATAAGAGACAG GGAGAGGAAACGGGGTCAAT	GTCTCGTGGGCTCGGAGATGTGTATAAGAGACAG ATAAGCAGGAGTCAGTGGGG
PAH1 OT4	TCGTCGGCAGCGTCAGATGTGTATAAGAGACAG ATTCTGCTGCTTTCCACCTG	GTCTCGTGGGCTCGGAGATGTGTATAAGAGACAG TTGGCAAGTCTCCTCTGTCC
PAH1 OT5	TCGTCGGCAGCGTCAGATGTGTATAAGAGACAG TCCCACCTTCACATCATCTGA	GTCTCGTGGGCTCGGAGATGTGTATAAGAGACAG GATTCCCCAAGGCAGTGATG
PAH1 OT6	TCGTCGGCAGCGTCAGATGTGTATAAGAGACAG CATGGAAAGGCAGAAGAGGC	GTCTCGTGGGCTCGGAGATGTGTATAAGAGACAG TCAAGACCAAAATGCCTGCC
PAH1 OT7	TCGTCGGCAGCGTCAGATGTGTATAAGAGACAG TTCTGTAGTTCTTTCTGTTCTCA	GTCTCGTGGGCTCGGAGATGTGTATAAGAGACAG ACATCTACAATGGTGGCACT
PAH1 OT8	TCGTCGGCAGCGTCAGATGTGTATAAGAGACAG TGAGTTCCTAAATTCCCAGTTGC	GTCTCGTGGGCTCGGAGATGTGTATAAGAGACAG AGCTTTTGTCTTTCAGAAATTCA
PAH1 OT9	TCGTCGGCAGCGTCAGATGTGTATAAGAGACAG GGCAGGTTGACATAAGAGCC	GTCTCGTGGGCTCGGAGATGTGTATAAGAGACAG TGTAGCCTCTGATGTTGCCA
PAH1 OT10	TCGTCGGCAGCGTCAGATGTGTATAAGAGACAG GGCTTGTCACCCACTCTCTT	GTCTCGTGGGCTCGGAGATGTGTATAAGAGACAG GAAGGTTCAGGCAGGCTTTT
PAH1 OT11	TCGTCGGCAGCGTCAGATGTGTATAAGAGACAG TGTAATTCAATTTAACCAGCGCT	GTCTCGTGGGCTCGGAGATGTGTATAAGAGACAG CCAGTAGATGCCCTTAATTCCC
PAH1 OT12	TCGTCGGCAGCGTCAGATGTGTATAAGAGACAG TGGGAGCATGGAAGTACAAGA	GTCTCGTGGGCTCGGAGATGTGTATAAGAGACAG TCACATTCAAGAAGCTTGCAAA
PAH1 OT13	TCGTCGGCAGCGTCAGATGTGTATAAGAGACAG CCCTACTTTGGTGCTGTGC	GTCTCGTGGGCTCGGAGATGTGTATAAGAGACAG ACTCAATGGCTCTCAGAATATGT
PAH1 OT14	TCGTCGGCAGCGTCAGATGTGTATAAGAGACAG AGGGATCAAATAGGAAATTTCGA	GTCTCGTGGGCTCGGAGATGTGTATAAGAGACAG ACTCTAGGAAGACACAGCTCT
PAH1 OT15	TCGTCGGCAGCGTCAGATGTGTATAAGAGACAG CAACAATCTGGCCTTAAACTGC	GTCTCGTGGGCTCGGAGATGTGTATAAGAGACAG TGAGAATGAGAAGGCAGCCA
PAH1 OT16	TCGTCGGCAGCGTCAGATGTGTATAAGAGACAG TGTTTCTCTTTGTGTACCTCAGT	GTCTCGTGGGCTCGGAGATGTGTATAAGAGACAG ACAAGGTTTCAGTTCACAAAGC
PAH1 OT17	TCGTCGGCAGCGTCAGATGTGTATAAGAGACAG CTCCCTGTGGCTGAGCTG	GTCTCGTGGGCTCGGAGATGTGTATAAGAGACAG GGTTCTTGGGTGTGACTCTG
PAH1 OT18	TCGTCGGCAGCGTCAGATGTGTATAAGAGACAG GTTTCTCTTTGTGTACCTCAGT	GTCTCGTGGGCTCGGAGATGTGTATAAGAGACAG ACTTGGCCCATATTTTCCCT
PAH1 OT19	TCGTCGGCAGCGTCAGATGTGTATAAGAGACAG GCTTGGCAAATATTTAGTACCCA	GTCTCGTGGGCTCGGAGATGTGTATAAGAGACAG TCTGGAGGTGGTGCAAGATT
PAH1 OT20	TCGTCGGCAGCGTCAGATGTGTATAAGAGACAG CGTGGCCCAAAAGAATGACA	GTCTCGTGGGCTCGGAGATGTGTATAAGAGACAG AGTTGAGCCTGTGATGGGAC
PAH1 OT21	TCGTCGGCAGCGTCAGATGTGTATAAGAGACAG TCCAATGGTCTTTTGATTGTGT	GTCTCGTGGGCTCGGAGATGTGTATAAGAGACAG AGCACCTGGATCTGATTGTG
PAH1 OT22	TCGTCGGCAGCGTCAGATGTGTATAAGAGACAG ACACCACATTGACTCTTCTCTT	GTCTCGTGGGCTCGGAGATGTGTATAAGAGACAG CCTCCCCAGCCATGTAGATC
PAH1 OT23	TCGTCGGCAGCGTCAGATGTGTATAAGAGACAG AGACGCTTCTGTGGAGACC	GTCTCGTGGGCTCGGAGATGTGTATAAGAGACAG CCTTTCTCACACACAGACTCC
PAH1 OT24	TCGTCGGCAGCGTCAGATGTGTATAAGAGACAG ACAAACACCATTCCCAGCAG	GTCTCGTGGGCTCGGAGATGTGTATAAGAGACAG CCACCTCCATGTCTGCTTTT
PAH1 OT25	TCGTCGGCAGCGTCAGATGTGTATAAGAGACAG CCATGTTTAGAAGGGGAGAATGA	GTCTCGTGGGCTCGGAGATGTGTATAAGAGACAG TGGTCATGTGTTTTAGGGGAA
PAH1 OT26	TCGTCGGCAGCGTCAGATGTGTATAAGAGACAG ATTTCTTAGGGCTGTCATAACA	GTCTCGTGGGCTCGGAGATGTGTATAAGAGACAG TCAAAAGAAGCACAGCCCTG

PAH1 OT27	TCGTCGGCAGCGTCAGATGTGTATAAGAGACAG TGGTTTGAGCAGAAAGTCGC	GTCTCGTGGGCTCGGAGATGTGTATAAGAGACAG TGTCCTCCATGTCCCTTGTC
PAH1 OT28	TCGTCGGCAGCGTCAGATGTGTATAAGAGACAG TCTTCTGCATAACAAAATGTCA	GTCTCGTGGGCTCGGAGATGTGTATAAGAGACAG ACAAAGACAGGGTGCAATAC
PAH1 OT29	TCGTCGGCAGCGTCAGATGTGTATAAGAGACAG AAGTAAAGTAAGTTGGAGAGGCA	GTCTCGTGGGCTCGGAGATGTGTATAAGAGACAG GTAGAAAGGTAAGTGGCTCGG
PAH1 OT30	TCGTCGGCAGCGTCAGATGTGTATAAGAGACAG AAGGAAAACCTATGCGCTGC	GTCTCGTGGGCTCGGAGATGTGTATAAGAGACAG TCACTGCTGCTAAGTACCCT
PAH1 OT31	TCGTCGGCAGCGTCAGATGTGTATAAGAGACAG TTGTCCAAGGTCACACAGCT	GTCTCGTGGGCTCGGAGATGTGTATAAGAGACAG TTTCTGAGATTGGGTGGTGC
PAH1 OT32	TCGTCGGCAGCGTCAGATGTGTATAAGAGACAG TAGAGCAGTGAACGGGAGAG	GTCTCGTGGGCTCGGAGATGTGTATAAGAGACAG GACTGGCTCTGTCCCATCAT
PAH1 OT33	TCGTCGGCAGCGTCAGATGTGTATAAGAGACAG TCCCTTATTCCATAGGCACCA	GTCTCGTGGGCTCGGAGATGTGTATAAGAGACAG TGGAAACCAACACCATACCAT
PAH1 OT34	TCGTCGGCAGCGTCAGATGTGTATAAGAGACAG AGGAAACCAAACTCTCCCCT	GTCTCGTGGGCTCGGAGATGTGTATAAGAGACAG CCAGAGAGGGAAAGAACACAC
PAH1 OT35	TCGTCGGCAGCGTCAGATGTGTATAAGAGACAG CAAGGCTGGGAGAGAAGGAG	GTCTCGTGGGCTCGGAGATGTGTATAAGAGACAG GTTAGGGAGAAGACTGGGCT
PAH1 OT36	TCGTCGGCAGCGTCAGATGTGTATAAGAGACAG CTTGTAAGGCAGGAACCACA	GTCTCGTGGGCTCGGAGATGTGTATAAGAGACAG CCAGGGTCACATCACAAAGC
PAH1 OT37	TCGTCGGCAGCGTCAGATGTGTATAAGAGACAG TCACTTACTCCAGGAAGCCC	GTCTCGTGGGCTCGGAGATGTGTATAAGAGACAG GCAAATCCCTGAGGTGGTCA
PAH1 OT38	TCGTCGGCAGCGTCAGATGTGTATAAGAGACAG TGAGCCCATAATTTGCGGAAG	GTCTCGTGGGCTCGGAGATGTGTATAAGAGACAG CGATTGAGCTGGATGTGAGG
PAH1 OT39	TCGTCGGCAGCGTCAGATGTGTATAAGAGACAG GGTGAAAACAGAAGACAGGCA	GTCTCGTGGGCTCGGAGATGTGTATAAGAGACAG AGGGTCCTTCAATTCTACCTCT
PAH1 OT40	TCGTCGGCAGCGTCAGATGTGTATAAGAGACAG TGTTAACCAAAACCAAAGCACT	GTCTCGTGGGCTCGGAGATGTGTATAAGAGACAG GCAATTGTGAAAATTATGCCTGC
PAH1 OT41	TCGTCGGCAGCGTCAGATGTGTATAAGAGACAG TGCTGTGGTATGCTCATTGA	GTCTCGTGGGCTCGGAGATGTGTATAAGAGACAG CTTGAAGTGATTTAAGTGAGCCT
PAH1 OT42	TCGTCGGCAGCGTCAGATGTGTATAAGAGACAG CACAGAAGGAATGTACACCCC	GTCTCGTGGGCTCGGAGATGTGTATAAGAGACAG ATGGCTCCTAATATTCGGGG
PAH1 OT43	TCGTCGGCAGCGTCAGATGTGTATAAGAGACAG AGTAAGCAGCATTTCCCAAACA	GTCTCGTGGGCTCGGAGATGTGTATAAGAGACAG CTCTGGCAAGGGTTTTGAAC
PAH1 OT44	TCGTCGGCAGCGTCAGATGTGTATAAGAGACAG TGCAAACTGTATCTTCGATTTT	GTCTCGTGGGCTCGGAGATGTGTATAAGAGACAG TGTACACCCAAGCTGACGTT
PAH1 OT45	TCGTCGGCAGCGTCAGATGTGTATAAGAGACAG TCTACTACACCACACTTGCAGA	GTCTCGTGGGCTCGGAGATGTGTATAAGAGACAG TGGTGACTATTATCCCTCCTGT
PAH1 OT46	TCGTCGGCAGCGTCAGATGTGTATAAGAGACAG ATAGCCACCCTAAAGACCGC	GTCTCGTGGGCTCGGAGATGTGTATAAGAGACAG ACACACACACACACACAAGCAT
PAH1 OT47	TCGTCGGCAGCGTCAGATGTGTATAAGAGACAG CTGTGCCAGCTTCTCAGACT	GTCTCGTGGGCTCGGAGATGTGTATAAGAGACAG GGAAGACAAACCAAAATTGAGGA
PAH2 OT1	TCGTCGGCAGCGTCAGATGTGTATAAGAGACAG TGCTGCAGGGTAAACAAAACA	GTCTCGTGGGCTCGGAGATGTGTATAAGAGACAG ACAGGGTTTTGCTATGCTGC
PAH2 OT2	TCGTCGGCAGCGTCAGATGTGTATAAGAGACAG GGGCTTGATTTGTGTCTGCT	GTCTCGTGGGCTCGGAGATGTGTATAAGAGACAG TGCCTCTCCTCATTGCATCA
PAH2 OT3	TCGTCGGCAGCGTCAGATGTGTATAAGAGACAG GAGGCACAGAGAGGCAGAG	GTCTCGTGGGCTCGGAGATGTGTATAAGAGACAG CGCATGTCTGTGTAGCTATGT
PAH2 OT4	TCGTCGGCAGCGTCAGATGTGTATAAGAGACAG TCCCTTATTCCATAGGCACCA	GTCTCGTGGGCTCGGAGATGTGTATAAGAGACAG TGGAAACCAACACCATACCAT
PAH2 OT5	TCGTCGGCAGCGTCAGATGTGTATAAGAGACAG TGCTGTGGTATGCTCATTGA	GTCTCGTGGGCTCGGAGATGTGTATAAGAGACAG CTTGAAGTGATTTAAGTGAGCCT
PAH2 OT6	TCGTCGGCAGCGTCAGATGTGTATAAGAGACAG AGCTTCTGGTAGTCTGGTGG	GTCTCGTGGGCTCGGAGATGTGTATAAGAGACAG GGTAGGTCTCTTCAAGCCACT
PAH2 OT7	TCGTCGGCAGCGTCAGATGTGTATAAGAGACAG CACACACACACACGCACG	GTCTCGTGGGCTCGGAGATGTGTATAAGAGACAG TGTTTCTTTGATCCCTAAGCACT
PAH2 OT8	TCGTCGGCAGCGTCAGATGTGTATAAGAGACAG ACTGGTATAAAACTATGGTGTGT	GTCTCGTGGGCTCGGAGATGTGTATAAGAGACAG CGGAGTGTGGGGTGAAAATC
PAH2 OT9	TCGTCGGCAGCGTCAGATGTGTATAAGAGACAG ACACCATAGACCACGCTGAG	GTCTCGTGGGCTCGGAGATGTGTATAAGAGACAG TCAGTCCAGTTCCTCAAATTCA

PAH2 OT10	TCGTCGGCAGCGTCAGATGTGTATAAGAGACAG ATTCTTGACTTCTGTGTACCCA	GTCTCGTGGGCTCGGAGATGTGTATAAGAGACAG AAAGTGGTTTCGTAGGCTGG
PAH2 OT11	TCGTCGGCAGCGTCAGATGTGTATAAGAGACAG TCCTTTAAAGCACAGTGGTACT	GTCTCGTGGGCTCGGAGATGTGTATAAGAGACAG TGATCAACAAGAGACAGGGCT
PAH2 OT12	TCGTCGGCAGCGTCAGATGTGTATAAGAGACAG ACAATTGTGGGTGAGATGCA	GTCTCGTGGGCTCGGAGATGTGTATAAGAGACAG CTCTGAGCTTTCCATGCACG
PAH2 OT13	TCGTCGGCAGCGTCAGATGTGTATAAGAGACAG AGGGTTCAGGAGCAGCTG	GTCTCGTGGGCTCGGAGATGTGTATAAGAGACAG TCAGTGCCCGGAAATGATTAA
PAH2 OT14	TCGTCGGCAGCGTCAGATGTGTATAAGAGACAG TTGGCTTTGGAGGGGCTAAT	GTCTCGTGGGCTCGGAGATGTGTATAAGAGACAG GCTCTCTCCACCTTCAACCT
PAH2 OT15	TCGTCGGCAGCGTCAGATGTGTATAAGAGACAG TTTCTAGGGCACAGGTCTCC	GTCTCGTGGGCTCGGAGATGTGTATAAGAGACAG CGGTAATGCAAGTGATGGGG
PAH2 OT16	TCGTCGGCAGCGTCAGATGTGTATAAGAGACAG GACGAACACTTCAGAACATTACA	GTCTCGTGGGCTCGGAGATGTGTATAAGAGACAG CAAGTGCTGTTGGTGAAAGGA
PAH2 OT17	TCGTCGGCAGCGTCAGATGTGTATAAGAGACAG ACATAGATGCAATTGGAAGCGA	GTCTCGTGGGCTCGGAGATGTGTATAAGAGACAG CTAACCCACACCTCTCTCCC
PAH2 OT18	TCGTCGGCAGCGTCAGATGTGTATAAGAGACAG AGTAAGTTGGAGAGGCAGCC	GTCTCGTGGGCTCGGAGATGTGTATAAGAGACAG GCATGAACCTCTGTGTCCG
PAH2 OT19	TCGTCGGCAGCGTCAGATGTGTATAAGAGACAG TGTGTACACTCGTTACATATGGT	GTCTCGTGGGCTCGGAGATGTGTATAAGAGACAG AGAAACAAATGCAACTTTCTGT
PAH2 OT20	TCGTCGGCAGCGTCAGATGTGTATAAGAGACAG TTACTTTATGTGCCATCATTCTG	GTCTCGTGGGCTCGGAGATGTGTATAAGAGACAG AGTTACTTAGAAACACTACTGCT
PAH2 OT21	TCGTCGGCAGCGTCAGATGTGTATAAGAGACAG TGCAAAGCCATCACTCTCAT	GTCTCGTGGGCTCGGAGATGTGTATAAGAGACAG CATCTCTGGCCCCTTCTCAA
PAH2 OT22	TCGTCGGCAGCGTCAGATGTGTATAAGAGACAG GGGGCCTAACAATGAATGACA	GTCTCGTGGGCTCGGAGATGTGTATAAGAGACAG CCTTGAATCATCTGGCTCCA
PAH2 OT23	TCGTCGGCAGCGTCAGATGTGTATAAGAGACAG AGAGTGTTCTTAGATCATCTGCA	GTCTCGTGGGCTCGGAGATGTGTATAAGAGACAG GCATTGCCAAACTGTTCTCTAC
PAH2 OT24	TCGTCGGCAGCGTCAGATGTGTATAAGAGACAG TCAATTTAACCAGCGCTGTCA	GTCTCGTGGGCTCGGAGATGTGTATAAGAGACAG ATTCCCTTCTCCCATTCCCC
PAH2 OT25	TCGTCGGCAGCGTCAGATGTGTATAAGAGACAG CCTCTTAGGGTTGTTGTGAGAA	GTCTCGTGGGCTCGGAGATGTGTATAAGAGACAG AGGAGTCTTAGCCTTGCCTG
PAH2 OT26	TCGTCGGCAGCGTCAGATGTGTATAAGAGACAG AGCACCTTAATTTCTGAGTCCT	GTCTCGTGGGCTCGGAGATGTGTATAAGAGACAG TGCCCTTCAGAAACGCAATT
PAH2 OT27	TCGTCGGCAGCGTCAGATGTGTATAAGAGACAG AGTCAGGAAACAACAGATGCT	GTCTCGTGGGCTCGGAGATGTGTATAAGAGACAG CCAGTAATGGGATTTTGGGGTC
PAH2 OT28	TCGTCGGCAGCGTCAGATGTGTATAAGAGACAG AGCAGTGTTAAGAATGGACTAGT	GTCTCGTGGGCTCGGAGATGTGTATAAGAGACAG CAGCTGATGTCTTAATGTTAACA
PAH2 OT29	TCGTCGGCAGCGTCAGATGTGTATAAGAGACAG GCATTCTTTACTGAGGGCCC	GTCTCGTGGGCTCGGAGATGTGTATAAGAGACAG CCTTCCCAGACTCTCCATCC
PAH2 OT30	TCGTCGGCAGCGTCAGATGTGTATAAGAGACAG CCGAGAAACACCGAAGAATGA	GTCTCGTGGGCTCGGAGATGTGTATAAGAGACAG CCTGAGGAGAGATGAACTGAGA
PAH2 OT31	TCGTCGGCAGCGTCAGATGTGTATAAGAGACAG CAGAAGCTGCAAAGTCCACA	GTCTCGTGGGCTCGGAGATGTGTATAAGAGACAG CTACCACCTCCATGTCTGCT
PAH2 OT32	TCGTCGGCAGCGTCAGATGTGTATAAGAGACAG TAACTCACAGCTGGCTCTCA	GTCTCGTGGGCTCGGAGATGTGTATAAGAGACAG GGCCCCACTAAGTCAGGATT
PAH2 OT33	TCGTCGGCAGCGTCAGATGTGTATAAGAGACAG TCCACAGTCTGTACAAGCACT	GTCTCGTGGGCTCGGAGATGTGTATAAGAGACAG TCTGCTAAGACCAATCAAAGGG
PAH2 OT34	TCGTCGGCAGCGTCAGATGTGTATAAGAGACAG CAGGTTGGAAGTTCACAAGGT	GTCTCGTGGGCTCGGAGATGTGTATAAGAGACAG TCCTGCACACTCCTGAGATG
PAH2 OT35	TCGTCGGCAGCGTCAGATGTGTATAAGAGACAG ACCTCAGTTCTTGGCTTCTG	GTCTCGTGGGCTCGGAGATGTGTATAAGAGACAG AGGGTCTTCGTGCTGTGTG
PAH2 OT36	TCGTCGGCAGCGTCAGATGTGTATAAGAGACAG ACAAAACCCCTGCTACACATAC	GTCTCGTGGGCTCGGAGATGTGTATAAGAGACAG GTGCCTGTGTGCGTGTGT
PAH2 OT37	TCGTCGGCAGCGTCAGATGTGTATAAGAGACAG GCTCAGTCTGGGTGCAATG	GTCTCGTGGGCTCGGAGATGTGTATAAGAGACAG ACCCTAGCTCTCTTTTCACTTCT
PAH2 OT38	TCGTCGGCAGCGTCAGATGTGTATAAGAGACAG GATGGACATGGATGCAGCTG	GTCTCGTGGGCTCGGAGATGTGTATAAGAGACAG GGCACTGAGAAGAGTACCCA
PAH2 OT39	TCGTCGGCAGCGTCAGATGTGTATAAGAGACAG CCTGGGTGACAAGAGCAAAA	GTCTCGTGGGCTCGGAGATGTGTATAAGAGACAG TGGTGAGTTAAGTTTGTCTGAA

PAH2 OT40	TCGTCGGCAGCGTCAGATGTGTATAAGAGACAG GCTAAAGGAACCAGGACTTAGG	GTCTCGTGGGCTCGGAGATGTGTATAAGAGACAG CATCAGTGCAGCCGTGTTG
PAH2 OT41	TCGTCGGCAGCGTCAGATGTGTATAAGAGACAG AGCACTCATAAATCAACAACAAA	GTCTCGTGGGCTCGGAGATGTGTATAAGAGACAG TGAGATGGTGGGAGAATGTGT
PAH2 OT42	TCGTCGGCAGCGTCAGATGTGTATAAGAGACAG GAGCCAAGCCTTCTCATGAA	GTCTCGTGGGCTCGGAGATGTGTATAAGAGACAG CAGCGGTAGTGTGGTGGA
PAH2 OT43	TCGTCGGCAGCGTCAGATGTGTATAAGAGACAG CTGGCGAATAAGACCCCTCA	GTCTCGTGGGCTCGGAGATGTGTATAAGAGACAG TAATGCAAGAGACCGTCCCA
PAH2 OT44	TCGTCGGCAGCGTCAGATGTGTATAAGAGACAG GCAAACGAAAGATAGCACCG	GTCTCGTGGGCTCGGAGATGTGTATAAGAGACAG GAAGGGGCATCTGAGACACT
PAH2 OT45	TCGTCGGCAGCGTCAGATGTGTATAAGAGACAG GGCTTGTCACCCACTCTCTT	GTCTCGTGGGCTCGGAGATGTGTATAAGAGACAG TGGGCTTAAACACAACTGTCC
PAH2 OT46	TCGTCGGCAGCGTCAGATGTGTATAAGAGACAG GGATCAGTAGCCCATAAGAACG	GTCTCGTGGGCTCGGAGATGTGTATAAGAGACAG TGTCCTGGTCTCACTCTCCA
PAH2 OT47	TCGTCGGCAGCGTCAGATGTGTATAAGAGACAG GCCAAACTATTCCCACCTTCA	GTCTCGTGGGCTCGGAGATGTGTATAAGAGACAG TGGCAGGGAGAACAGATGAT
