# Supplementary material for: Rapid and definitive treatment of phenylketonuria in variant-humanized mice with corrective editing
Source: Nat Commun. 2023 Jun 10;14:3451. doi: 10.1038/s41467-023-39246-2 (PMC10257655; doi:10.1038/s41467-023-39246-2)
Supplement: Supplementary file 3 — Reporting Summary [file 41467_2023_39246_MOESM3_ESM.pdf]

nature portfolio

Corresponding author(s): Kiran Musunuru, Mohamad-Gabriel Alameh, Xiao Wang

Last updated by author(s): May 26, 2023

Reporting Summary

Nature Portfolio wishes to improve the reproducibility of the work that we publish. This form provides structure for consistency and transparency in reporting. For further information on Nature Portfolio policies, see our Editorial Policies and the Editorial Policy Checklist.

Statistics

For all statistical analyses, confirm that the following items are present in the figure legend, table legend, main text, or Methods section.

n/a	Confirmed	
☐	☒	The exact sample size (*n*) for each experimental group/condition, given as a discrete number and unit of measurement
☐	☒	A statement on whether measurements were taken from distinct samples or whether the same sample was measured repeatedly
☒	☐	The statistical test(s) used AND whether they are one- or two-sided<br>*Only common tests should be described solely by name; describe more complex techniques in the Methods section.*
☒	☐	A description of all covariates tested
☒	☐	A description of any assumptions or corrections, such as tests of normality and adjustment for multiple comparisons
☐	☒	A full description of the statistical parameters including central tendency (e.g. means) or other basic estimates (e.g. regression coefficient) AND variation (e.g. standard deviation) or associated estimates of uncertainty (e.g. confidence intervals)
☒	☐	For null hypothesis testing, the test statistic (e.g. *F*, *t*, *r*) with confidence intervals, effect sizes, degrees of freedom and *P* value noted<br>*Give P values as exact values whenever suitable.*
☒	☐	For Bayesian analysis, information on the choice of priors and Markov chain Monte Carlo settings
☒	☐	For hierarchical and complex designs, identification of the appropriate level for tests and full reporting of outcomes
☒	☐	Estimates of effect sizes (e.g. Cohen's *d*, Pearson's *r*), indicating how they were calculated

Our web collection on statistics for biologists contains articles on many of the points above.

Software and code

Policy information about availability of computer code

Data collection	GraphPad Prism v9.5.0
Data analysis	GraphPad Prism v9.5.0, CRISPResso2 v2.0.31, Cas-Designer v1.2

For manuscripts utilizing custom algorithms or software that are central to the research but not yet described in published literature, software must be made available to editors and reviewers. We strongly encourage code deposition in a community repository (e.g. GitHub). See the Nature Portfolio guidelines for submitting code & software for further information.

Data

Policy information about availability of data

All manuscripts must include a data availability statement. This statement should provide the following information, where applicable:

- Accession codes, unique identifiers, or web links for publicly available datasets
- A description of any restrictions on data availability
- For clinical datasets or third party data, please ensure that the statement adheres to our policy

DNA sequencing data that support the findings of this study have been deposited in the NCBI Sequence Read Archive under accession codes PRJNA976718 [https://www.ncbi.nlm.nih.gov/bioproject/976718] and PRJNA976729 [https://www.ncbi.nlm.nih.gov/bioproject/976729]. All other data supporting the findings of this study (Figs. 1–4) are available within the Article and its Supplementary Information. The GRCh38 Ensembl v98 reference genome (ftp://ftp.ensembl.org/pub/release-98/fasta/homo_sapiens/dna/Homo_sapiens.GRCh38.dna.chromosome.{1-22,X,Y,MT}.fa, ftp://ftp.ensembl.org/pub/release-98/fasta/homo_sapiens/dna/Homo_sapiens.GRCh38.dna.nonchromosomal.fa) annotation was used. Source data are provided with this paper.

Human research participants

Policy information about studies involving human research participants and Sex and Gender in Research.

Reporting on sex and gender	N/A
Population characteristics	N/A
Recruitment	N/A
Ethics oversight	N/A

Note that full information on the approval of the study protocol must also be provided in the manuscript.

Field-specific reporting

Please select the one below that is the best fit for your research. If you are not sure, read the appropriate sections before making your selection.

☒ Life sciences ☐ Behavioural & social sciences ☐ Ecological, evolutionary & environmental sciences

For a reference copy of the document with all sections, see nature.com/documents/nr-reporting-summary-flat.pdf

Life sciences study design

All studies must disclose on these points even when the disclosure is negative.

Sample size	Sample sizes were determined based on ethical considerations, i.e., using the minimum number of breeding pairs to yield the minimum number of animals needed to reproducibly establish normalization of blood phenylalanine levels and non-zero whole-liver editing (2 to 4 mice for each experimental arm).
Data exclusions	No data were excluded.
Replication	Experiments were repeated or duplicated at least once, with the exception of the individual mouse studies for which parameters were changed in order to ensure the robustness of the results (age of mice, genotypes, guide RNA) and the liver histology study. All attempts at replication were successful.
Randomization	Randomization was used when feasible for mouse experiments. Randomization was not used for cellular experiments because in each experiment the various samples were on the same plates and exposed to identical conditions.
Blinding	Although the investigators responsible for group allocation were not blinded to the allocation scheme--due to the PKU hypopigmentation phenotype making it unavoidably obvious which mice had PKU and which did not, and which mice had normalization of the phenotype after treatment--in general the investigators responsible for analyses were blinded to the allocation scheme.

Reporting for specific materials, systems and methods

We require information from authors about some types of materials, experimental systems and methods used in many studies. Here, indicate whether each material, system or method listed is relevant to your study. If you are not sure if a list item applies to your research, read the appropriate section before selecting a response.

Materials & experimental systems

n/a	Involved in the study
☒	☐ Antibodies
☐	☒ Eukaryotic cell lines
☒	☐ Palaeontology and archaeology
☐	☒ Animals and other organisms
☒	☐ Clinical data
☒	☐ Dual use research of concern

Methods

n/a	Involved in the study
☒	☐ ChIP-seq
☒	☐ Flow cytometry
☒	☐ MRI-based neuroimaging

Eukaryotic cell lines

Policy information about cell lines and Sex and Gender in Research

Cell line source(s)	HuH-7 cells (RRID:CVCL_0336) were obtained directly from the Japanese Collection of Research Bioresources (JCRB) Cell Bank.
Authentication	Upon generation of the PAH P281L homozygous HuH-7 cell line, authentication of the cell line was performed via confirmation of the presence of the P281L variant in the homozygous state. No additional authentication with respect to identity as HuH-7 cells was performed.
Mycoplasma contamination	The cell line was not tested for mycoplasma contamination.
Commonly misidentified lines (See ICLAC register)	No commonly misidentified lines were used in this study.

Animals and other research organisms

Policy information about studies involving animals; ARRIVE guidelines recommended for reporting animal research, and Sex and Gender in Research

Laboratory animals	The humanized PAH P281L PKU mouse model (Mus musculus, C57BL/6J strain) was generated by the Penn Vet Transgenic Mouse Core and used for experiments at 4 weeks of age or 8 weeks of age. Wild-type C57BL/6J mice used for breeding were obtained from The Jackson Laboratory (stock no. 000664). Mice were maintained on a 12-hour light/12-hour dark cycle, with a temperature range of 65°F to 75°F and a humidity range of 40% to 60%, and were fed ad libitum with a chow diet (LabDiet, Laboratory Autoclavable Rodent Diet 5010).
Wild animals	No wild animals were used in the study.
Reporting on sex	Mice of both sexes were used, although allocation to groups did not take sex into account due to the lack of significant effect of sex on blood phenylalanine levels. Due to the small sample sizes chosen in part due to ethical considerations (using the minimum number of animals needed for experimentation) and the finding that all treated mice, without exception, experienced substantial reductions in blood phenylalanine levels, sex-based analysis was not performed.
Field-collected samples	No field-collected samples were used in the study.
Ethics oversight	All procedures used in animal studies were approved by the Institutional Animal Care and Use Committee at the University of Pennsylvania (protocol #805887), where the studies were performed, and were consistent with local, state, and federal regulations as applicable, including the National Institutes of Health Guide for the Care and Use of Laboratory Animals.

Note that full information on the approval of the study protocol must also be provided in the manuscript.
